# Phylogenetic Analysis and Antibiotics Resistance of *Listeria Monocytogenes* Contaminating Chicken Meat in Surabaya, Indonesia

**DOI:** 10.1155/2020/9761812

**Published:** 2020-03-01

**Authors:** Eduardus Bimo Aksono, Katty Hendriana Priscilla Riwu, A. T. Soelih Estoepangestie, Herinda Pertiwi

**Affiliations:** Department of Basic Veterinary Medicine, Department of Veterinary Public Health, Faculty of Veterinary Medicine, Institute of Tropical Disease, Department of Health, Faculty of Vocational Studies, Airlangga University, Surabaya, Jawa Timur 60115, Indonesia

## Abstract

The objective of this study was to identify the phylogenetic analysis and antibiotic resistance of *Listeria monocytogenes* contaminating chicken meat in Surabaya. 60 chicken meat samples were collected from supermarkets, mobile vendors, and traditional markets in Surabaya. A selective medium is used for isolation and identification of *Listeria monocytogenes* by chopping 25 grams of the chicken meat and to put it into the sterilized Erlenmeyer flasks. Some methods were used for the identification procedures, such as biochemical and morphological tests, antibiotic resistance test, PCR, and sequencing; also a phylogenetic analysis was conducted by a neighbor-joining analysis using Genetix Mac ver 8.0 with hlyA genes of *Listeria monocytogenes* recorded in GenBank, such as Lineage I (KC808543), Lineage II (AY229462, AY229346, AY229499, and AY229404), Lineage III (KJ504139, HQ686043, KJ504116, and DQ988349), and Lineage IV (EU840690, EF030606). The result shows that the prevalence of *L. monocytogenes* in Surabaya contaminating the chicken meat samples from the supermarkets was 10% (2/20), from the mobile vendors was 0/20 (0%), and from the traditional markets was 5% (1/20). It was seen from the band at 456 bp fragment. Furthermore, three isolates found in Surabaya were included in the new lineages which were resistant to old-generation antibiotics such as sulfamethonazole-trimetophrim (SXT) and amoxyllin sulbactam (MAS), but they were still sensitive to new-generation antibiotics such as cefotaxime (CTX) and meropenem (MEM).

## 1. Introduction

The most popular animal protein source in Indonesia is chicken meat. It is cheap, delicious, and easy to cook; also, various dishes can be made of it. In 2018, Indonesia produced 2,144,013 tons of broiler meat; it increased then on 2016 by only 1,905,497.28 tons and the demand grows fast every year [1]. One of the biggest producers of broiler chicken in Indonesia is the East Java province. The harvest was collected in Surabaya as the capital city of East Java and also the biggest city in Indonesia after Jakarta before it is distributed to other cities and widely bought by customers from supermarkets, traditional markets, and mobile vendors.

With the increasing demand for chicken meat, people's anxiety for food-borne diseases also arises. One of the agents causing it is *Listeria monocytogenes* [2]. This bacterium can be found anywhere in food, water, soil, vegetables, animals, and humans. In addition, it also has high potential to infect humans and animals with high mortality rates [3]. Human listeriosis has been reported in the USA being caused by consuming cantaloupe, smoked fish, marinated products, meat products, and vegetables which are contaminated with *L. monocytogenes* [4, 5].

According to Janzten et al. [6], *Listeria* genus is categorized as gram-positive bacterium that contains 6 species of *Listeria*: *Listeria monocytogenes, Listeria innocua, Listeria seeligeri, Listeria welshimeri, Listeria ivanovii*, and *Listeria grayi.* One of them known as the most pathogenic to humans is *L. monocytogenes* which consists of 4 lineages (lineages I, II, III, and IV) [7]. These bacteria can grow at the temperature of 1°C–45°C and they can proliferate at cold or freezing temperatures.

Fast food such as nonpasteurized meat and milk products stored for a long time at 4°C is a potential source of *L*. *monocytogenes* infection. Sometimes, *L. monocytogenes* can also be found in processed food products. *L. monocytogenes* contamination after food being processed is a critical point for human health. Therefore, considerable knowledge is needed so that the prevention of *L. monocytogenes* bacteria transmission in the environment or in food products from livestock and its dairy products can be done appropriately. Furthermore, a fast and accurate detection technique for the presence of *L. monocytogenes* in food is needed so that the infected individuals can be immediately treated [8]. Rodriguez et al. [9] also reported that the products most infected with *L. monocytogenes* are poultry and beef meat stored in refrigerator; smoked and fresh meat. Human listeriosis caused by *L. monocytogenes* is an intermittent disease with mild-to-severe flu symptoms as well as meningitis and septicemia manifestation. The group at risk are pregnant women and immunocompromised people. In pregnant women, it can lead to abortion, premature birth, and birth defects.

In Indonesia, food poisoning due to *Listeria* sp. infection, especially *L. monocytogenes*, is less frequent than from *E. Coli* and *Salmonella* bacteria. *L. monocytogenes* contamination in Indonesia has not been widely reported as in developed countries [10]. In Malaysia, it is reported that various local food sold by street vendors is ready-to-eat (RTE) food. High prevalence of pathogens is found in this kind of food. It is also found in raw food and RTE products sold in hypermarkets, although their hygiene is assumed to be better, but pathogens of foodborne disease and *L. monocytogenes* are also identified [11].

Typically, *L. monocytogenes* is susceptible to a wide range of antibiotics, although some isolates have been reported resistant to many antibiotics [12]. Some of the virulence markers of *L. monocytogenes* such as listeriolysin O (encoded by the hlyA gene) have a role in regulating virulence and pathogenicity [13]. Even in food, bacteria originated from environment can result in the expression of varied virulence genes that will result in different infection levels [16].

According to Harsoyo and Andini [10], Indonesian National Standards have actually established that food products of animal origin in Indonesia should not contain *Listeria* sp. bacteria, as well as in US and Europe. The Food and Agricultural Organization (FAO) guidelines also emphasize proactive and risk-based modern food security system. Therefore, it is necessary to identify diseases affecting the population and the presence of pathogens in food and to establish risk mitigation measures [15].

The objective of this study was to identify phylogenetic analysis and the antibiotic resistance of *L. monocytogenes* contaminating chicken meat in Surabaya, Indonesia, especially for Ampicillin, amoxylin sulbactam, cefotaxime, meropenem, and sulfamethonazole-trimetophrim which are commonly used by broiler farmers as antibiotic growth promoters and therapeutic antibiotics in Indonesia [16].

## 2. Materials and Methods

### 2.1. Study Design, Study Area, and Sampling

A cross-sectional prospective study was carried out in Surabaya metropolitan city. 60 raw chicken meat samples were collected from supermarkets, traditional markets, and mobile vendors as the main meat suppliers for Surabaya people, which included 20 samples from each place. Simple random sampling technique was employed for all the samples that were collected aseptically from randomly selected supermarkets, traditional markets, and mobile vendors in Surabaya. The meats were placed in sterile leakproof container in cold chain box. The samples were transported to the Tropical Disease Diagnosis Center (TDDC), Institute of Tropical Disease, Airlangga University.

### 2.2. Biochemical and Morphological Test

A selective medium is used for isolation and identification of *L. monocytogenes*. 25 grams of the chicken meat was put into the sterilized Erlenmeyer flasks, and 225 ml Buffered Listeria Enrichment was added and then homogenized using a vortex mixer for 2 minutes and incubated at 30°C for 24–28 hours [17]. After incubation, identification was conducted by taking bacterial suspension from the Erlenmeyer flasks with a transfer loop and then grazing it to PALCAM medium and then it was incubated at 37°C for 24–48 hours. The colony of *L. monocytogenes* on green PALCAM agar medium was surrounded by black zone [17]. The positive samples undergone blood medium hemolysis test at 37°C for 24 hours. It was conducted to see the forming *β*-hemolysis. Gram staining processing showed purple or violet color in the microscopic observation [17]. And then, Sulfide Indole Motility (SIM) test was done to identify sulfide, indole, and bacterial movement after being incubated for at 37°C for 24 hours [17]. Triple sugar iron test (TSIA) and glucose test were conducted to identify fermentation of glucose, lactose, and sucrose [17]. Methyl Red-Voges Proskauer (MR-VP) was performed to show pink color and the formation of acetyl-methyl carbinol [17]. The confirmation test (CAMP test) was used to identify umbrella-like bacterial growth due to excessive hemolytic zone around the grazed *Staphylococcus aureus* and *Rhodococcus equi* [17].

### 2.3. Antibiotics Resistance Test

The prepared pure culture was taken by sterilized cotton swab and spread over the surface of Muller Hinton Agar (MHA) and left for 5 minutes. Paper disc containing antibiotics was put on MHA with pure culture separated 25–30 nm and then incubated at 35°C for 24 hours [18]. The antibiotics sensitivity test was measured based on the inhibition diameter used vernier calipers. The antibiotics tested were Ampicillin, amoxylin sulbactam, Cefotaxime, Meropenem, and sulfamethonazole-trimetophrim and then the result was interpreted using the inhibition zone table from the National Community for Clinical Laboratory Standard (NCCLS) [17].

### 2.4. *Listeria monocytogenes* Detection with PCR

DNA extraction of *L. monocytogenes* was conducted with Kit Qiagen. 5 *μ*l suspension from the extraction was used directly as template for CR amplification of hlyA gene fragments. 20 *μ*l PCR reaction consisted of 12.5 *μ*l master mix, 0.5 *μ*l distilled water, 0,5 *μ*l forward primer (F), 0.5 *μ*l reverse primer (R), and 5 *μ*l DNA template. The primers used were F: 5′-GCAGTTGCAAGCGCTTGGAGTGAA-3′ and R: 5′-GCA-ACGTATCCTCCAGAGTGATCG-3′ [19]. PCR conditions included predenaturation 95°C for 5 minutes, denaturation 95°C for 30 seconds, annealing 54.6°C for 30 seconds, extension 72°C for 1 minute 30 seconds, and final extension 72°C for 5 minutes. There were 35 cycles of PCR process. 5 *μ*l PCR product was put into 2% electrophoresis gel and then used in electrophoresis medium of 100 volts for 60 minutes. After the electrophoresis, gel was then taken for observation with UV light. DNA target fragments' visualization was shown at 456 bp with UV transilluminator [19].

### 2.5. Sequencing and Phylogenetic Analysis

#### 2.5.1. PCR Product Purifying Result Using Low-Melting Agarose Methods

PCR product was acquired and then purified. The steps were as follows: (1) 2% agarose gel was prepared for L Agarose (Low-Melting Agarose) using ethidium bromide 1 mg/ml; (2) DNA from the PCR result about 5 *μ*l was added with loading buffer for 1 *μ*l. During application, the mixture was poured into every other slot. After the electrophoresis, the result was seen with UV light with 365 nm wavelength. The DNA band was then cut with a cutter (while being beamed with UV light, the cutter must always be washed after cutting every DNA band); (3) the piece of Agarose gel mixed with a DNA slice was inserted into 2 ml microtube; (4) the DNA in the gel was then purified with reagent QIAquick PCR purification kit from Qiagen by following the instructions attached in the kit.

#### 2.5.2. Pure DNA Labelling

The result of the purifying was then labelled through prosequencing PCR process using hlyA gene primers (forward). In this procedure, the labelling was done using labelled dideoxynucleotide triphosphate dye, BigDye Termination Kit V.1.1 from Applied Biosystems. The condition of prosequencing PCR was 1 cycle of predenaturation at 96°C for 3 minutes, followed by 25 cycles which covered denaturation phase at 96°C for 10 seconds, annealing phase at 50°C for 5 seconds, and extension phase at 60°C for 4 minutes.

#### 2.5.3. Labelled DNA Precipitation

DNA product from the prosequencing PCR was then precipitated with these steps: (1) 150–200 *μ*l labelled DNA was put into the new microtube and then precipitated using ethanol with addition of 1/10x NaAc 3M (pH 5.2) volume for 15 *μ*l and 2X ethanol 100% volume for 300 *μ*l, mixed with the vortex mixer and kept at –20°C for 30 minutes; (2) centrifugation was done at 14,000 rpm for 10 minutes; then the supernatant was discarded carefully; (3) the pellet was washed with 550 *μ*l 70% ethanol and then centrifuged at 12,000 rpm for 5 minutes and the supernatant was discarded carefully; (4) the pellet was dried with vacuum pump for 10 minutes and resuspended with 10 *μ*l of TE pH 8 solution; and (5) the DNA was kept at –20°C and ready for sequencing procedures.

#### 2.5.4. Nucleotide Sequencing

After the DNA was purified using low-melting Agarose and labelled with hlyA primer gene, there was a sequencing procedure to get the nucleotide formation. The nucleotide sequence was determined with BigDye Terminator Cycle Sequencing kit, EDTA Hi-Di formamide, and special 0.5 ml tube from Applied Biosystems and also an automatic sequencing device, ABI Prism 310 Genetic Analyzer.

#### 2.5.5. Phylogenetic Analysis

Nucleotide model profile acquired from *L. monocytogenes* isolates in Surabaya was then analyzed phylogenetically using a neighbor-joining analysis using Genetix Mac Ver 8.0 with hlyA genes from *L. monocytogenes* recorded in GenBank: Lineage I (KC808543), Lineage II (AY229462, AY229346, AY229499, and AY229404), Lineage III (KJ504139, HQ686043, KJ504116, and DQ988349), and Lineage IV (EU840690, EF030606).

## 3. Results and Discussion

**Figure 1:**
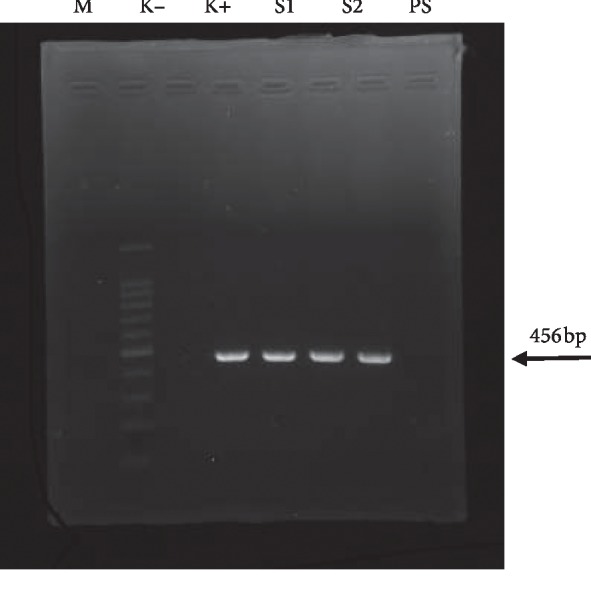
In 2% electrophoresis gel agar, the PCR result on *L. monocytogenes* showed contaminated chicken meat in Surabaya with 456 bp nucleotide length (M: marker; K+: positive control; K–: negative control; S1: sample from supermarkets; S2: sample from mobile vendors; and PS: sample from traditional markets).

In Figure 1, the overall prevalence of *L. monocytogenes* in Surabaya contaminating the chicken meat samples from the supermarkets was 10% (2/20), from the mobile vendors was 0/20 (0%), and from the traditional markets was 5% (1/20). It was seen from the band at 456 bp fragment.

**Figure 2:**
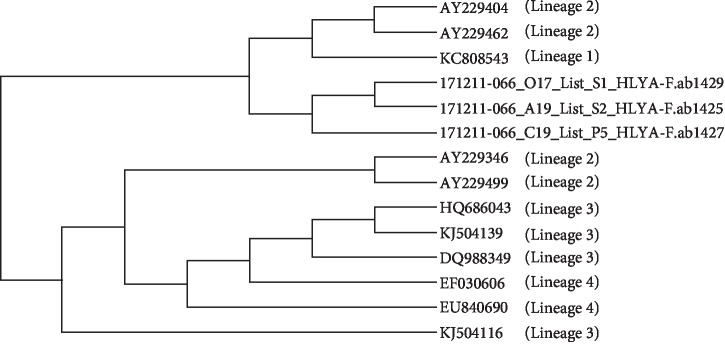
Phylogenetic analysis of selected strains of *L. monocytogenes* from different sources, representing the four distinct lineages, based on the listeriolysin (*hlyA*) gene. The Gene Bank accession numbers of the isolates used are given.

In Figure 2, from the phylogenetic analysis, compared to the 4 lineages recorded in GenBank, there were 3 isolates from Surabaya, Indonesia, which showed distinct lineages.

From Table 1, we can see 3 isolates of *L. monocytogenes* contaminating the chicken meat collected from the supermarkets or traditional markets in Surabaya, Indonesia, have been resistant to sulfamethonazole-trimetophrim (SXT) and amoxyllin sulbactam (MAS), but they were still sensitive to cefotaxime (CTX) and meropenem (MEM).

This study was not linear with that of Sugiri et al. [20] that showed that *L. monocytogenes* contamination found in supermarket was about 10% and traditional markets about and mobile vendors was 0% in Bandung City, Indonesia. However, it was also in line with the previous study which reported that *L. monocytogenes* prevalence found in chicken meat ranged between 15 and 35%, where the bacteria grew well in chicken meat which was kept at 0–8°C temperature without having been vacuumed and in 10 days the bacteria reached 10^8^–10^9^ cells/gram [8, 18, 21].

Ampicillin, amoxyllin sulbactam, cefotaxime, meropenem, and sulfamethonazole-trimetophrim are the most widely used antibiotic drugs in broiler farms in Indonesia as feed additive and growth promoter; they increase production and increase the efficiency of feed use [16]. This is associated with a high prevalence of respiratory infections, paratyphoid fever, pharyngitis, tonsillitis, varicella, typhoid fever, and tuberculosis during the 2008–2009 period in human case due to antibiotic resistance reaction [22].

The presence of antibiotics residues in chicken meat beyond maximum permissible limits is a matter of serious concern. Heat treatments can decrease the risk of some antibiotics groups but do not guarantee the complete degradation of these antibiotic residues present in meat. Some of the developed countries, including Denmark, Norway, Sweden, and the European Union, have restricted the application of antibiotics for growth-promoting purposes. Training farmers to monitor withdrawal periods, banning the use of antibiotics as preventive treatment, and adopting the veterinary feed and drugs regulation are important parameters to mitigate the emergence of antibiotic resistance in bacteria related to broiler production [23].

Personal hygiene of chicken meat seller in mobile vendors and traditional markets in Surabaya is very poor because none of them wear masks and gloves. Rafikah et al. [24] explain that all personnel who work in the food processing should maintain hygiene; for example, they must wear clean clothes and equipment, apron, cap, masks, and gloves to minimize bacterial contamination of food product.

Although meat storage in supermarkets is better and cleaner than traditional markets in Surabaya, the condition of ready-to-eat foods (RTE) storage and display in supermarkets allows *L. monocytogenes* to grow and thrive in food even at frozen temperatures. Chen et al. [25] reported that *L. monocytogenes* was found in the food storage refrigerators, either edible food with little or no preheating process, so it can be a harmful threat for consumer. The presence of *L. monocytogenes* in chicken meat in traditional markets, mobile vendors, and supermarkets in Surabaya showed that during the production process, from the cutting to storage, *L. monocytogenes* contamination occurred; therefore, strict monitoring and preventive procedures are needed minimize it.

Antunes et al. [26] and Srinivasan et al. [27] explained that *L. monocytogenes* isolates from poultry meat and dairy product exhibited resistance to one or more antibiotics, indicating that animal products are potential carrier for antibiotic-resistant foodborne diseases. *Listeria monocytogenes* is naturally susceptible to *β*-lactams group antibiotic such as penicillin amoxicillin, meropenem, and ampicillin, or combined with an aminoglycoside (gentamicin) as standard therapy for human listeriosis [28, 29]. *β*-lactams against *L. monocytogenes* inhibit the synthesis of bacterial cell wall peptidoglycan [30]. For patients who are allergic to lactams antibiotic, sulfamethoxazoletrimethoprim can be used [31]; however, Srinivasan et al. [27] and Yucel et al. [32] have reported that this antibiotic is resistant to *L. monocytogenes* isolated from meat and dairy farm product.

Resistant microbes strains that emerged owing to antibiotic misdose can tolerate the effect of antibiotics at inhibitory concentration levels [33]. Consumption of meat contaminated with such resistant strains after improper processing or mismanagement enhances the chances of their transmission in humans [34]. *L. monocytogenes* may transfer resistant genes to human natural microbes through mutation, plasmid mediation (self-transferable plasmids and mobilizable plasmids), conjugative transposons, and efflux pumps [35–38]. The presence of such resistant strains can reduce the effectiveness of antibiotics used to treat infected individuals [39].

Vazquez-Boland et al. [40] proved that there was correlation between the virulence level and the strain type from the bacteria isolated; the clinical origin has a lower virulence than the origin of the food. To ensure the presence of *L. monocytogenes* in the original environment, identification for one major virulence factor is a better choice. Among the various virulence factors, LLO (58 kDa hemolysin protein encoded by the hlyA gene) is a major virulence factor and pathogenic marker for detecting *Listeria* sp. The phylogenetic study of *L. monocytogenes* is essential to improve our understanding on how *L. monocytogenes* is transmitted from animals or the environment through food to humans. The results showed that all three isolates contaminating chicken meat in Surabaya formed distinctive lineages, as they were not included in 4 previously reported lineages [7].

In the present study, the result of antibiotic resistance test showed that the three isolates from Surabaya, Indonesia, that contaminated chicken meat in supermarkets, mobile vendors, and traditional markets were resistant to some old-generation antibiotics such as sulfamethonazole-trimetophrim (SXT) and amoxyllin sulbactam (MAS), but they were still sensitive to new-generation antibiotics such as cefotaxime (CTX) and meropenem (MEM).

Meropenem in monotherapy has been found active against intracellular *L. monocytogenes* infection on meningitis case in the experimental study; it was 10-fold more potent than ampicillin and ertapenem [41]. Meropenem has bacteriostatic effect after 5 hours of treatment and starts bactericidal effect after 24 hours [42]; it could initiate bacterial severe cell damages [41].

Cefotaxime is a bactericidal derived from cephalosporin; it has a broad-spectrum activity against gram-positive microorganisms and exceptional activity against most gram-negative microorganisms by interfering with synthesis of their cell walls. It is widely considered to be the antibiotic of choice for the management of neonatal meningitis and sepsis caused by gram-negative bacteria including *L. monocytogenes* [43]. Cefotaxime has received wide acceptance as a first-line antibiotic for many infections in neonates, infants, and children. Cefotaxime caused a significant enhancement of IL-2 production by cells and increased the secretion of TNF-*α*by peripheral blood mononuclear cells; it is also suggested that it may modify the host immune response. Cefotaxime is a safe and effective antibiotic in treating meningitis bacterial infections, of which 80% of these microorganisms are resistant to ampicillin [43].

This result was different from previous studies showing that *Listeria* sp. is generally still sensitive to all antibiotics [44]. It cannot be separated from the habit of inappropriate use of antibiotics in livestock industry and communities in some developing countries including Indonesia. Therefore, efforts to raise awareness on the importance of the correct use of antibiotics regarding the dose and duration should be a concern of both government and communities.

## 4. Conclusion

The contamination of *L. monocytogenes* in chicken meat in supermarkets, mobile vendors, and traditional markets in Surabaya indicates the potential listeriosis distribution. Moreover, the three isolates found were included as distinctive lineages and already resistant to old-generation antibiotics such as sulfamethonazole-trimetophrim (SXT) and amoxyllin sulbactam (MAS). Therefore, surveillance policies for potential food contamination and antibiotic sensitivity of *L. monocytogenes* are required, while also ensuring effective antibiotic treatment.

## Figures and Tables

**Table 1:** Result from antibiotic resistance test of *L. monocytogenes* (present study) isolates from chicken meat in Surabaya, Indonesia.

AntiobiticAntibiotic	Isolate S1	Isolate S2	Isolate P5
Ampicillin (AML)	Intermediate	Intermediate	Intermediate
Amoxyllin Ssulbactam (MAS)	Resistant	Resistant	Resistant
Cefotaxime (CTX)	Sensitive	Sensitive	Sensitive
Meropenem (MEM)	Sensitive	Sensitive	Intermediate
Sulfamethonazole-trimetophrim (SXT)	Resistant	Resistant	Resistant

S1: sample from supermarkets, S2: sample from mobile vendors, and PS1: sample from traditional markets.

## Data Availability

The data used to support the findings of this study are available from the corresponding author upon request.
